# Clinical Lectures on Diseases of the Heart and Aorta

**Published:** 1898-12

**Authors:** 


**Clinical Lectures on Diseases of the Heart and Aorta.** By George William Balfour, M.D. Third Edition. Pp. xxi., 479. London: Adam and Charles Black. 1898.

The third edition of these valuable lectures has appeared twenty-two years after the publication of the first. It requires considerable energy of mind and character for a physician who has so extensive a medical career as Dr. Balfour to re-edit old lectures and bring them as nearly as possible up to date.

We consider that Dr. Balfour's book is not merely of historic interest, but a lucid exposition of the leading features of heart disease that may be read with profit both by students and by those comparatively well-versed in this branch of medicine. Possibly the most interesting chapter, to those who are not seeking elementary information, is that dealing with murmurs audible over the pulmonary area. As is well known, Dr. Balfour has been a strong supporter of Naunyn's theory that the so-called hæmic murmur heard over the pulmonary area is due to mitral regurgitation, the *bruit* produced by such regurgitation being thought to be conducted by the appendix of the left auricle to the chest wall. This explanation is, of course, open to criticism. Dr. Balfour, however, refers also to cases of loud murmurs heard in the same region not due to anæmia or stenosis of the pulmonary artery. Records of examples are given, and the causation of the murmur discussed. Dr. Balfour seems inclined to adopt Quincke's theory, which attributes the murmur to a somewhat abnormal position of the pulmonary artery, owing to retraction of the left lung and to compression of the artery by the heart during systole. The lumen of the vessel is altered and a *bruit* produced.

In angina pectoris Dr. Balfour strongly advocates chloroform, if nitrite of amyl fails to give relief. He considers that it is a perfectly safe remedy to leave in the hands of the patient. Some chloroform may be put in the bottom of a tumbler, or a smelling-bottle may be filled. When used by the sufferer from an attack, the tumbler or smelling-bottle drops from the nose as soon as narcosis approaches. In the chapter on the therapeutics of heart disease great stress is laid upon arsenic as a cardiac tonic, and it is considered to be of particular value in cases of angina pectoris, where its effect is said to be occasionally marvellous. For increasing the flow of urine in ordinary cases of heart disease diuretin is recommended.

